# A new pathway for neuroprotection against tau hyperphosphorylation via δ-opioid receptor initiated inhibition of CDK5 and AMPK signaling

**DOI:** 10.3389/fnagi.2025.1587219

**Published:** 2025-06-24

**Authors:** Jiahui Li, Yuan Xu, Gianfranco Balboni, Ying Xia

**Affiliations:** ^1^Department of Aeronautics and Astronautics, Shanghai Key Laboratory of Acupuncture Mechanism and Acupoint Function, Fudan University, Shanghai, China; ^2^Shanghai Research Center for Acupuncture and Meridians, Shanghai, China; ^3^Department of Neurosurgery, The First People’s Hospital of Changzhou, Changzhou, China; ^4^Clinical Medical Research Center, The Third Affiliated Hospital of Soochow University, Changzhou, China; ^5^Department of Life and Environmental Sciences, University of Cagliari, Cagliari, Italy

**Keywords:** Alzheimer’s disease, AMPK, CDK5, δ-opioid receptor, neuroprotection, tau hyperphosphorylation

## Abstract

**Introduction:**

Alzheimer’s disease (AD) is a progressive neurodegenerative disease characterized by decreased memory and cognitive impairment. Abnormal tau hyperphosphorylation ultimately forms neurofibrillary tangles, which is one of the most important pathological features of AD. Since we have previously shown that the δ-opioid receptor (DOR) is neuroprotective in the brain, we asked if DOR plays any role in the control of tauopathy.

**Methods:**

In the PC12 cell model with okadaic acid-induced tau hyperphosphorylation, cell viability and cytotoxicity were evaluated by using CCK8 assay kit and lactate dehydrogenase cytotoxicity assay kit. The techniques of western blot and immunofluorescence were used to investigate the effect of DOR on tau hyperphosphorylation.

**Results:**

We found that DOR activation inhibited okadaic acid-induced tau hyperphosphorylation in PC12 cells and attenuated the cell cycle reactivation and apoptosis. The DOR effect was blocked by Naltrindole, a DOR antagonist. Furthermore, the mechanistic studies showed that the DOR displayed its effect by reducing the expression of cyclin-dependent kinase (CDK) 5 and AMP-activated protein kinase (AMPK) in the model of tauopathy.

**Discussion:**

Our novel findings suggest that DOR signaling may protect neurons from AD injury by inhibiting tau hyperphosphorylation.

## 1 Introduction

Alzheimer’s disease (AD) is a progressive neurodegenerative disease. Current treatment of AD is very limited although more and more individuals worldwide are affected by the serious disorder. It is of utmost importance to search for new solutions for AD treatment. The existing evidence suggests that multiple cellular and molecular mechanisms are involved in its pathogenesis, including accumulation of amyloid beta (Aβ), formation of neurofibrillary tangles (NFTs), excessive neuroinflammation, mitochondrial damage, synaptic loss, and neuron re-entry into the cell cycle (Reddy et al., 2012; Frade and Ovejero-Benito, 2015; Hou et al., 2021; Putthanbut et al., 2024). One of the most important pathological features of AD is intracellular NFTs caused by tau hyperphosphorylation (Scheltens et al., 2021).

Tau protein is a major microtubule-associated protein present in neurons, and its biological function is to promote microtubule aggregation and stabilize microtubule structure (Avila et al., 2004; Brandt et al., 2005). Its hyperphosphorylation makes tau to lose biological function, dissociate from microtubules, easily aggregate into paired helical filaments, and become toxic molecules, causing microtubule structure collapse and neuronal degeneration (Reddy, 2011; Kshirsagar et al., 2021). Several protein kinases and protein phosphatases, including cyclin-dependent kinase (CDK5), AMP-activated protein kinase (AMPK), glycogen synthase kinase 3β (GSK3β), mitogen-activated protein kinase (MAPK), and protein phosphatase 2A (PP2A), etc., are involved in the control of tau hyperphosphorylation. Therefore, targeting tau and the related molecules is of a great potential for treatment of tauopathy in AD (Vingtdeux et al., 2011; Wang and Mandelkow, 2016).

The re-entry of neurons into the cell cycle is also one of the main neuropathological features of AD (Keeney et al., 2012; Frade and Ovejero-Benito, 2015). Re-entering the cell cycle is considered abortive and triggers neuronal apoptosis rather than cell division (Beckmann et al., 2023). Early studies have shown that cell-cycle regulatory factors such as CyclinD1, CyclinB1, PCNA, and Cdc2 are abnormally expressed and activated in NFT-containing neurons (Andorfer et al., 2005; Bonda et al., 2010). Additional research further indicates that hyperphosphorylation of tau protein causes the neurons to re-enter the cell cycle, leading to neuronal death (Khurana et al., 2006). Therefore, inhibition of cell cycle reactivation is another way to attenuate neuronal injury induced by tauopathy.

Delta-opioid receptor (DOR) is a G protein-coupled receptor that is highly expressed in the central nervous system (Klenowski et al., 2015). Substantial evidence has shown that the activation of DOR plays an important role in neuroprotection, thus attenuating neurodegenerative injury (Chao and Xia, 2010; He et al., 2013; Xu et al., 2020a; Xu et al., 2020b; Xu et al., 2022). However, the role of DOR in AD is controversial (Tanguturi and Streicher, 2023). Teng et al. (2010) observed that activating DOR enhanced the activity of β-site-APP cleaving enzyme 1 (BACE1) and γ-secretase, increasing the production of Aβ in 293T cells. Using a more specific and potent DOR agonist in the neuron-like model (PC12 cells), we have recently shown that DOR activation decreased the expression and activity of BACE1, reducing the production of toxic Aβ (Xu et al., 2020a). Furthermore, we found that the activation of DOR reduced the deposition of Aβ plaques in the cortex and hippocampus and improved cognitive impairment in APP/PS1 mice (Xu et al., 2025). However, it is unknown if DOR plays any role in the control of tauopathy. In this work, we specifically asked if DOR signaling affects tau hyperphosphorylation and neuronal viability. Our findings from a tau cell model reveal that DOR activation effectively attenuated tau hyperphosphorylation and reduced neuron re-entry into the cell cycle and apoptosis through the regulation of CDK5 and AMPK pathways, suggesting that DOR signaling protects against tauopathy by inhibiting CDK5 and AMPK pathways.

## 2 Materials and methods

### 2.1 Cell culture and treatment

The highly differentiated rat PC12 cell line was purchased from the Type Culture Collection of the Chinese Academy of Sciences, Shanghai, China. The cells were cultured with high-glucose Dulbecco’s Modified Eagle Medium (Gibco) containing 10% fetal bovine serum (Sigma), and 1% penicillin/streptomycin (Gibco) in a 37°C incubator with 95% humidity and 5% CO2. The culture conditions of PC12 cells and their morphologic characteristics were the same as those of our previous studies (Xu et al., 2020a).

To induce tau hyperphosphorylation, PC12 cells were exposed to okadaic acid (OA) (HY-N6785, MCE). The cells were also treated with a specific DOR agonist UFP-512, which was synthesized by our team (Balboni et al., 2002; Chao et al., 2007; Kang et al., 2009; Cao et al., 2015; Xu et al., 2019; Xu et al., 2022), and naltrindole hydrochloride (N766608, Macklin).

### 2.2 Cell viability assay

The CCK8 assay kit (C0038, Beyotime) was used to evaluate the cell viability of OA on PC12 cells. Cells (3 × 10^3^ cells/well) were seeded into each well in a 96-well plate, the control group was that of the cells without OA treatment. After incubation overnight, different concentrations of (0, 30, 50, and 70 nM) OA were added. After 24 h of incubation, 10 μL CCK8 was added to each well. After 1.5 h of incubation, the absorbance at 450 nm was measured using a microplate reader.

### 2.3 Lactate dehydrogenase (LDH) release

Cell toxicity was evaluated by detecting the release of LDH in the culture medium using an LDH cytotoxicity assay kit (C0016, Beyotime). The experiment was conducted according to the manufacturer’s instructions. In brief, 24 h after treating PC12 cells with OA, 120 μL of culture medium was transferred from each well to a new 96-well plate, then 60 μL of reaction solution was added to each well. The plate was placed on a shaker at room temperature for 30 min. The release of LDH into the culture medium was measured by detecting the absorbance at 490 nm using a microplate reader.

### 2.4 Western blot analysis

RIPA lysate containing protease inhibitors (Roche) and phosphatase inhibitors (Roche) was used to lyse cells for 30 min. The protein samples were collected after centrifugation at 4°C, 12000 *g* for 20 min. Protein concentration was measured using the BCA protein assay kit. Then we performed 10%–12.5% gel electrophoresis for protein separation. Then proteins were transferred to the polyvinylidene fluoride (PVDF) membranes. After blocking at room temperature for 1 h with 5% skimmed milk, the membranes were incubated overnight at 4°C with the following primary antibodies as needed: P-Tau Thr231 (1:1000, ab151559, Abcam), Tau5 (1:1000, ab80579, Abcam), P-Tau Ser262 (1:1000, #11111, Signalway Antibody), P-GSK-3β Ser9 (1:2000, #9322, Cell Signaling Technology), GSK-3β (1:2000, #12456, Cell Signaling Technology), P-AMPK Thr172 (1:1000, #2535, Cell Signaling Technology), AMPK (1:2000, #2532, Cell Signaling Technology), Caspase3 (1:1000, #14220, Cell Signaling Technology), β-actin (1:2000, #4970, Cell Signaling Technology), CDK5 (1:2000, sc-6247, Santa Cruz), CyclinD1 (1:2000, sc-8396, Santa Cruz), and CyclinB1 (1:2000, sc-245, Santa Cruz). After then, the membranes were incubated with the corresponding secondary antibodies, i.e., goat anti-rabbit secondary antibody (1:2000, #7074, Cell Signaling Technology) and goat anti-mouse secondary antibody (1:2000, #7076, Cell Signaling Technology) at room temperature for 1 h. The immunoreactivity was detected by ECL and bands were visualized on the Bio-Rad system. Quantitative analysis of protein blot results was performed using ImageJ software.

### 2.5 Immunofluorescence (IF) staining

After fixing the cells with 4% paraformaldehyde at room temperature for 20 min, 1 mL of 0.5% Triton-100 was allowed to penetrate for 15 min. Then, the cells were blocked at room temperature with 5% BSA for 30 min and incubated overnight at 4°C with the P-Tau Thr231 (1:500, ab151559, Abcam) antibody. After incubating with the goat anti-rabbit secondary antibody, Alexa Fluor^®^ 488 conjugate (1:2000, A0423, Beyotime) at room temperature for 1 h, DAPI staining was performed for 5 min. The anti-quenching agent was dropped onto a slide for sealing and photographed under a fluorescence microscope.

### 2.6 Statistical analysis

For data analysis, all experiments were three independent experiments. The data are expressed as mean±SD. For multiple comparisons, data were analyzed by one-way ANOVA. *P* < 0.05 was considered statistically significant.

## 3 Results

### 3.1 DOR activation reduced tau hyperphosphorylation

**FIGURE 1:**
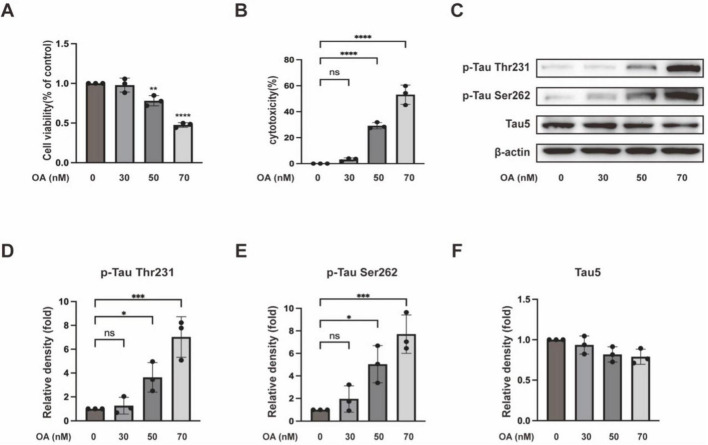
Okadaic acid (OA)-induced tau hyperphosphorylation. PC12 cells were treated with different concentrations of OA at 0, 30, 50, and 70 nM for 24 h. **(A)** Cell viability was detected through CCK8 assay. **(B)** Cell toxicity was detected through LDH release assay. **(C)** Western blot assay was used to detect tau hyperphosphorylation levels at the Thr231 and Ser262 sites and total tau. **(D–F)** Quantitative analysis of tau hyperphosphorylation at Thr231 and Ser262 sites, along with total tau protein levels, was performed using ImageJ. Band intensities were normalized to β-actin as the loading control. Statistical analysis was conducted using the one-way ANOVA test. The data (mean ± SD) were from three independent experiments. *P < 0.05, **P < 0.01, ***P < 0.001, ****P < 0.0001 vs. control. ns, no significance.

Okadaic acid is an inhibitor of protein phosphatase PP2A. Both *in vivo* and *in vitro* experiments have confirmed that OA induces tau hyperphosphorylation (Chou and Yang, 2021). We first tested the effect of OA on the PC12 cells in our system by measuring cell viability and cytotoxicity in the PC12 cells incubated with different concentrations of OA for 24 h. The CCK8 tests (Figure 1A) showed that 50 nM OA significantly decreased cell viability, and the LDH release assay (Figure 1B) showed that the same OA treatment markedly increased cytotoxicity. The immunoblotting detections (Figures 1C–E) showed that OA increased tau hyperphosphorylation in a concentration-dependent manner, while total tau remained unchanged (Figure 1F). Based on the dose-dependent tests, we chose OA with a concentration of 50 nM for the subsequent experiments.

**FIGURE 2:**
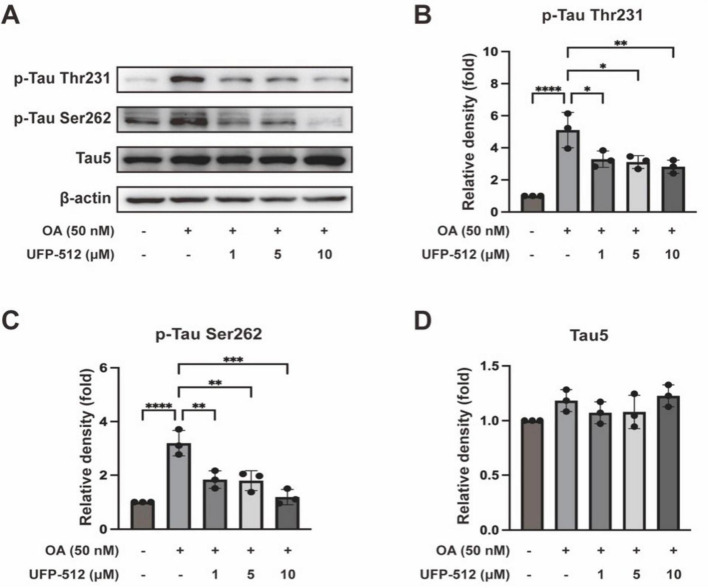
Delta-opioid receptor (DOR) activation decreased tau hyperphosphorylation. **(A)** PC12 cells were treated with OA (50 nM) and different concentrations (1, 5, 10 μM) of UFP-512 for 24 h. Western blot assay was used to detect the level of tau hyperphosphorylation at Thr231 and Ser262 sites as well as total tau. **(B–D)** Quantitative analysis of tau hyperphosphorylation at Thr231 and Ser262 sites, along with total tau protein levels, was performed using ImageJ. Band intensities were normalized to β-actin as the loading control. Statistical analysis was conducted using the one-way ANOVA multiple comparison analysis. The data (mean ± SD) were from three independent experiments. *P < 0.05, **P < 0.01, ***P < 0.001, ****P < 0.0001.

To investigate the effect of DOR activation on tau hyperphosphorylation, we used different concentrations (1, 5, 10 μM) of UFP-512, a specific DOR agonist in the OA-treated PC12 cells. As shown in Figures 2A–D, UFP-512 decreased the phosphorylation of tau protein at the Thr231 and Ser262 sites compared to that of the OA group, in a dose-response manner, while total tau levels were unchanged.

### 3.2 Inhibition of DOR reversed DOR’s protection

**FIGURE 3:**
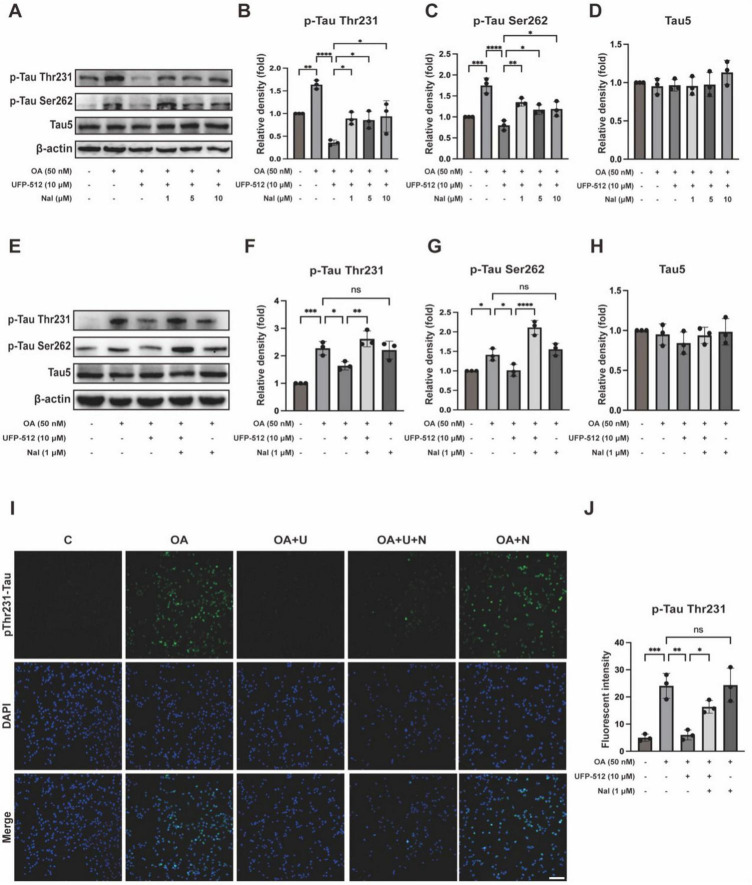
Delta-opioid receptor inhibitor reversed the inhibitory effect of DOR on tau hyperphosphorylation. **(A)** PC12 cells were treated with OA (50 nM), UFP-512 (10 μM), and naltrindole (1, 5, 10 μM) for 24 h. Western blot assay was used to detect the level of tau hyperphosphorylation at Thr231 and Ser262 sites and total tau. **(B–D)** Quantitative analysis of tau hyperphosphorylation at Thr231 and Ser262 sites, along with total tau protein levels, was performed using ImageJ. Band intensities were normalized to β-actin as the loading control. **(E)** PC12 cells were treated with OA (50 nM), UFP-512 (10 μM), and naltrindole (1 μM) for 24 h. Western blot assay was used to detect the level of tau hyperphosphorylation at Thr231 and Ser262 sites and total tau. **(F–H)** Quantitative analysis of tau hyperphosphorylation level at Thr231 and Ser262 sites, along with total tau protein levels, was performed using ImageJ. Band intensities were normalized to β-actin as the loading control. **(I)** Immunofluorescence staining of pThr231-Tau (green) and DAPI (blue) in the OA-treated PC12 cells of the control, OA, OA + U, OA + U + N and OA + N groups (scale bar = 100 μm). **(J)** Quantitative analysis of immunofluorescence intensity for tau phosphorylated at Thr231 was performed using ImageJ. Statistical analysis was conducted using the one-way ANOVA multiple comparison analysis. The data (mean ± SD) were from three independent experiments. *P < 0.05, **P < 0.01, ***P < 0.001, ****P < 0.0001. ns, no significance.

To ascertain the specific effect of DOR on tau, we investigated whether DOR inhibition affected the inhibitory effect of DOR on tau hyperphosphorylation. We added naltrindole, a DOR inhibitor, at different concentrations (1, 5, 10 μM) to the OA-treated PC12 cells. Western blot exhibited that naltrindole increased the level of phosphorylated tau protein at the Thr231 and Ser262 sites as compared to that of the OA + UFP-512 group. In contrast, the expression level of Tau5 had no significant changes (Figures 3A–D). The phosphorylation level of tau protein at the Thr231 and Ser262 sites was not significantly different between the OA and OA + naltrindole groups (Figures 3E–G), while the total tau has not changed (Figure 3H). The immunofluorescence staining results were consistent with that of western bolt (Figures 3I, J).

### 3.3 Potential mechanisms of DOR inhibition on tau phosphorylation

**FIGURE 4:**
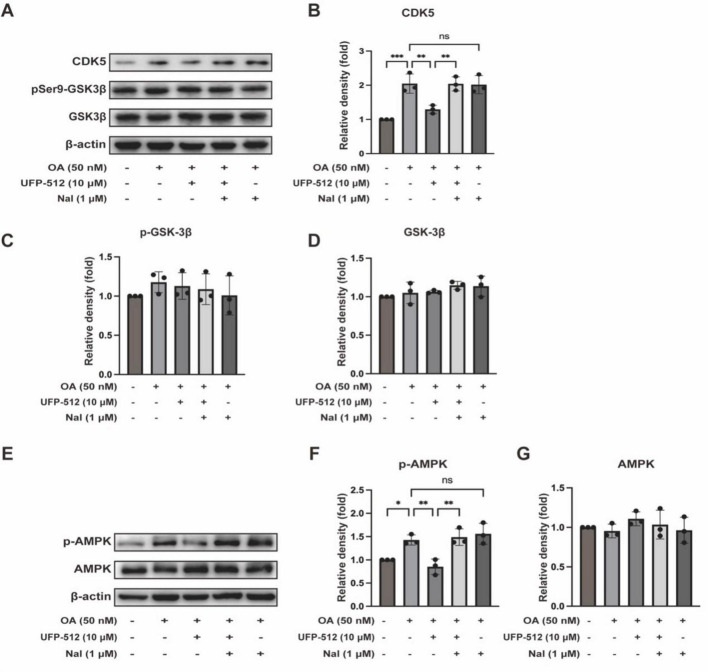
Delta-opioid receptor activation inhibited CDK5 and AMPK pathways. **(A,E)** PC12 cells were treated with OA (50 nM), UFP-512 (10 μM), and naltrindole (1 μM) for 24 h and then western blot assay with cell lysate was conducted. **(B–D,F,G)** Quantitative analysis of CDK5, p-GSK-3β, total GSK-3β, pAMPK, and total AMPK was performed using ImageJ. Band intensities were normalized to β-actin as the loading control. Statistical analysis was conducted using the one-way ANOVA multiple comparison analysis. The data (mean ± SD) were from three independent experiments. *P < 0.05, **P < 0.01, ***P < 0.001. ns, no significance.

CDK5 and GSK3β are two main kinases responsible for tau hyperphosphorylation (Engel et al., 2006; Kanungo et al., 2009). In our model, however, CDK5 significantly increased in response to the OA treatment, while GSK3β phosphorylation at Ser9 (inactive form) had no significant changes in the same model (Figure 4). Therefore, we further asked if DOR’s inhibition on tau hyperphosphorylation occurs by regulating CDK5. Figures 4A, B shows that the level of CDK5 was significantly increased in the OA group than in the control group, which was significantly reduced by DOR activation with UFP-512. The DOR effect could be reversed by the treatment of naltrindole. There was no appreciable difference between OA vs. OA + naltrindole groups. All these results suggest that DOR activation may exert an inhibitory effect on tau hyperphosphorylation via the CDK5 pathway.

AMPK is another kinase that phosphorylates tau. There is co-localization of AMPK and phosphorylated tau in the brain of AD patients (Vingtdeux et al., 2011). *In vitro* studies have demonstrated that AMPK activation also induces tau phosphorylation at multiple sites (Vingtdeux et al., 2011; Domise et al., 2016). Western blot results showed that AMPK phosphorylation was increased in the OA group. Our data showed that the addition of UFP-512 in the OA group inhibited the expression of pAMPK, while naltrindole reversed the UFP-512 action. Adding naltrindole alone to the OA group did not cause any significant change in pAMPK expression compared to the OA group (Figures 4E, F). Therefore, the AMPK pathway is likely involved in DOR action on tau hyperphosphorylation.

### 3.4 DOR activation inhibited neuronal cell cycle reactivation and neuronal apoptosis

**FIGURE 5:**
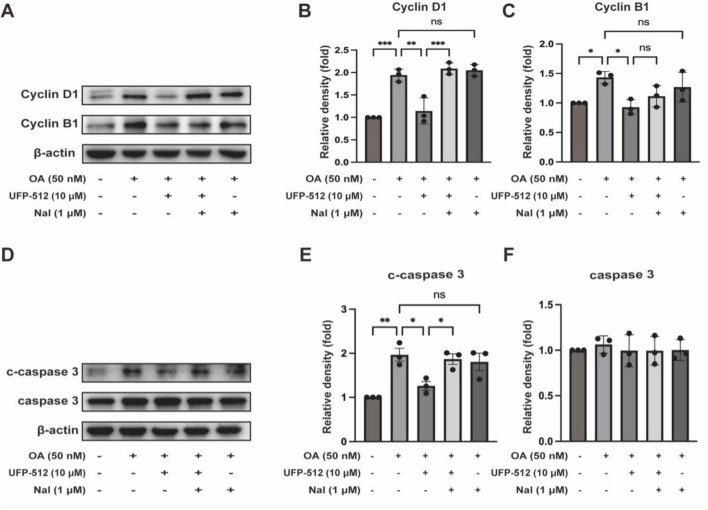
Delta-opioid receptor activation significantly ameliorated apoptosis and the expression of CyclinD1, and CyclinB1. **(A,D)** PC12 cells were treated with OA (50 nM), UFP-512 (10 μM), and naltrindole (1 μM) for 24 h, and then western blot assay was conducted with the cell lysate. **(B,C,E,F)** Quantitative analysis of CyclinD1, CyclinB1, c-caspase 3, and caspase 3 was performed using ImageJ. Band intensities were normalized to β-actin as the loading control. Statistical analysis was conducted using the one-way ANOVA multiple comparison analysis. The data (mean ± SD) were from three independent experiments. *P < 0.05, **P < 0.01, ***P < 0.001. ns, no significance.

Furthermore, we investigated whether DOR activation can inhibit cell cycle regulatory factors and reduce neuronal cell death, thus exerting a neuroprotective effect. We examined CyclinD1 and B1 as markers of G1 and G2 phases in the cell cycle progression, respectively. As shown in Figures 5A–C, the protein levels of CyclinD1 and CyclinB1 decreased with UFP-512 treatment. However, naltrindole increased the CyclinD1 level while CyclinB1 remained unchanged compared to OA + UFP-512 groups. Naltrindole alone had no effect on the same group. These results suggest that DOR activation may inhibit tau-induced cell cycle reactivation.

Moreover, we evaluated the level of apoptosis using the protein expression of cleaved caspase 3 and caspase 3. As shown in Figures 5D, E, the protein levels of cleaved caspase 3 decreased with UFP-512 treatment. Naltrindole inhibited the effect of UFP-512 in the OA-treated PC12 cells, while naltrindole alone had no significant effect on the same group. The expression of caspase 3 did not change in each group (Figure 5F). These results suggest that DOR activation inhibits tau-induced neuronal apoptosis.

## 4 Discussion

Tau hyperphosphorylation is one of the most important pathological features of AD. Therefore, several drugs targeting tau have been extensively studied, including tau gene silencing therapy, tau-based immunotherapy, regulation of tau post-translational modification (PTM), and reducing tau aggregation (Ossenkoppele et al., 2022). Unfortunately, most of them are terminated after clinical trials due to their toxicity or lack of efficacy (Ossenkoppele et al., 2022). Therefore, finding a new way to reduce tau hyperphosphorylation is of utmost importance for AD treatment. Toward this goal, we made the first finding that DOR signaling effectively inhibits tau hyperphosphorylation. Indeed, DOR activation markedly reduced tau hyperphosphorylation at the Thr231 and Ser262 sites, while DOR inhibition with naltrindole reversed such effect, which straightforwardly documented the DOR-mediated inhibition on tau hyperphosphorylation.

The imbalance between protein kinases and protein phosphatases is believed to be the main cause of tau hyperphosphorylation. CDK5 and GSK3β have been well-documented as key players in tau hyperphosphorylation (Lauretti et al., 2020; Onder et al., 2022; Li et al., 2023). CDK5 is a serine/threonine protein kinase, that is highly expressed in neurons and is essential for the basic function of neurons and neuronal migration (Ao et al., 2022). Reportedly, the level of CDK5 abnormally increased in the brain of AD patients (Lee et al., 1999). Its aberrant over-activation leads to hyperphosphorylation of tau at several sites including Thr231 and Ser262 (Chen et al., 2022). Inhibition of CDK5 led to a reduction in tau hyperphosphorylation in cortical neurons (Zheng et al., 2005). We found that DOR activation significantly reduced CDK5, suggesting that CDK5 is an important and specific target of DOR in the inhibition of tau hyperphosphorylation. GSK3β at Ser9 has been well studied as an inducing factor for tau hyperphosphorylation (Wei et al., 2020; Wang et al., 2022), but we did not see any significant change in GSK3β at Ser9 in our model. At present, however, we cannot rule out the role of GSK3β in tau hyperphosphorylation and DOR’s effect on GSK3β in our PC12 cell model because of the complexity of the GSK3β activity. In addition to Ser9 site, the activity of GSK3β can also be activated by the dephosphorylation of Ser389 and phosphorylation of Tyr216 (Muyllaert et al., 2008; Zhu et al., 2023). Moreover, PP2A can dephosphorylate GSK3β at Ser9 (Salcedo-Tello et al., 2011; Wang et al., 2015). Therefore, a deep exploration is needed to further clarify if DOR has a regulatory effect on GSK3β. Nevertheless, our comparative results between CDK5 and GSK3β clearly indicate the importance of CDK5 in the DOR inhibition of tau hyperphosphorylation. The activity of CDK5 is regulated by its activator p35. In AD, p35 undergoes calpain cleavage into p25, leading to abnormal activation of CDK5. The CDK5/p25 complex leads to tau hyperphosphorylation (Patrick et al., 1999; Lee et al., 2000). Therefore, we will detect the changes in p35 and p25 in future work to better understand DOR’s action on CDK5.

AMP-activated protein kinase is a cellular stress sensor that maintains energy homeostasis by controlling the activity of various metabolic enzymes. Some studies have shown that activation of AMPK is neuroprotective against neurodegenerative diseases (Neumann et al., 2021; Chen et al., 2023). For example, DOR activation leads to neuroprotection by elevating the level of AMPK to activate autophagy in the spinal cord injury (SCI) (Chen et al., 2023). However, others have shown that in certain situations (i.e., in convalescent ischemic stroke), AMPK activation promotes neuronal apoptosis (Liu et al., 2023). In the brains of patients with AD and other tauopathies, there was the accumulation of AMPK in pre-tangled and tangled-containing neurons (Vingtdeux et al., 2011). More recent studies showed that AMPK activation was associated with an increase in tau phosphorylation, whereas AMPK inhibition led to a decrease in tau phosphorylation and AMPK deficiency reduced tau pathology in the PS19 mice, indicating that AMPK activation preceded tau phosphorylation (Domise et al., 2016). Our data showed that AMPK was upregulated in OA-induced tau model, while DOR activation significantly decreased AMPK phosphorylation at the Thr172 site with a reduction of tau hyperphosphorylation, which could be reversed by DOR antagonist Naltrindole. Therefore, we believe that AMPK is another key target of DOR in the regulation of tau hyperphosphorylation. This finding raises several interesting questions about the underlying mechanisms. For example, a change in the expression and/or activity of AMPK upstream kinases (such as LKB1, CAMKK2, or TAK1) (Steinberg and Kemp, 2009) may be involved in the regulation. In addition, PP2A dephosphorylates AMPK at the Thr172 site (Ma et al., 2022), which may also be targeted by DOR signaling. We will further investigate these issues in next work.

Mammalian brain neurons are typically considered terminally differentiated cells and remain at rest in the G0 phase (Zhu et al., 2004). In the AD brain, an increase in CyclinD1 is considered the main regulatory factor that induces neuronal cells to enter G1 from the G0 phase (Zhu et al., 2004). Tau hyperphosphorylation upregulates CyclinD1, thus inducing hippocampal neurons to re-enter the S phase, thereby resulting neuronal apoptosis (Fang et al., 2016). Indeed, tau hyperphosphorylation co-localizes with CyclinB1 and CyclinD1 in primary hippocampal neurons (Wang et al., 2018). In the present work, DOR activation inhibited the expression of CyclinB1 and D1, suggesting a DOR-mediated protection via blocking cell cycle reactivation. However, it is necessary to comprehensively explore the role of DOR in cell cycle reactivation, such as its effect on the S phase. We also observed that DOR activation decreased cleaved caspase 3 in OA-induced PC12 cells. Since tau phosphorylation-induced cell-cycle activation in postmitotic neurons results in neuronal apoptosis *in vivo* (Khurana et al., 2006), we speculate that DOR activation exerts its inhibitory effect in the tau hyperphosphorylation model by inhibiting cell cycle reactivation, thus reducing neuronal cell apoptosis. However, it is vital to conduct a comprehensive study to deeply understand the relevant cellular processes based on our initial exploration. For example, our current work limited to the expression level of the apoptotic protein caspase 3 and its activated form for the evaluation of cell apoptosis. It is important to detect other molecular changes in apoptotic events, such as Bax and Bcl2, in next work for a better picture of the mechanisms.

Overall, our data suggest that DOR may be a promising target for better treatment of tauopathy in AD injury. To achieve this goal, it is important to further elucidate the mechanisms underlying the DOR-mediated inhibition on tau hyperphosphorylation, especially the precise cellular process of the DOR-mediated inhibition of CDK5 and AMPK. The equally important research is to dissect out the mechanisms for the DOR-mediated reduction of the cell cycle-related proteins and apoptotic proteins. Answers to these critical questions will better define the conditions and factors for a new therapy of tau-related injury in the brain.

However, we should keep in mind that the present data were obtained from a highly differentiated PC12 cell model. The PC12 cells originate from rat pheochromocytoma and differentiate into neuronal phenotypes under the stimulation of nerve growth factor (Westerink and Ewing, 2008). They have been widely used in research on neurodegenerative diseases such as AD (Li et al., 2020; Yong et al., 2024; Xie et al., 2025). Although they have physiological characteristic of nerve cells, they may differ in some aspects from “real” neurons, especially human neurons. Therefore, it is important to further validate our findings in human nerve cells (e.g., SH-SY5Y cells or iPSC-derived neurons) before more translational research.

## 5 Conclusion

**FIGURE 6:**
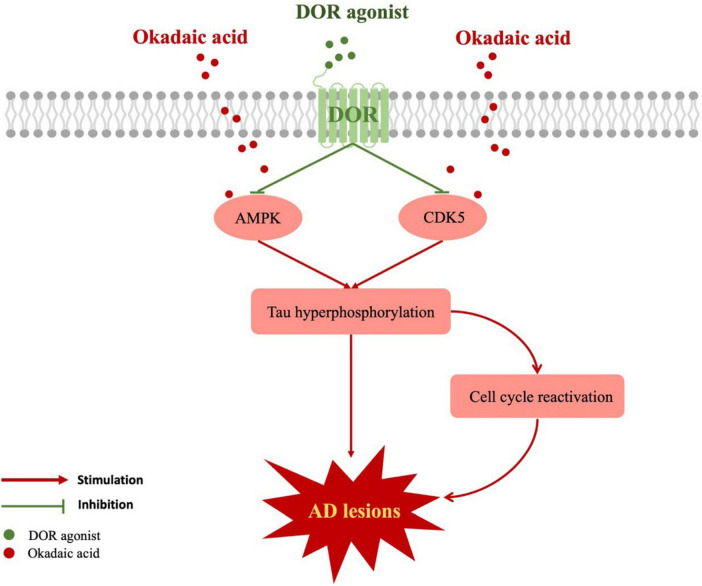
Schematic diagram of the mechanism for DOR against tauopathy. The red boxes represent the upregulated molecules when the cells were treated with okadaic acid. The red lines represent injurious effects, while the green lines indicate the inhibitory effects of DOR activation. Tau hyperphosphorylation could be stimulated by AMPK, CDK5, leading to cell cycle reactivation and causing AD lesions. DOR activation not only reduced tau hyperphosphorylation by inhibiting AMPK and CDK5, but also diminished cell cycle reactivation.

We first discovered a unique role of DOR in attenuating tau hyperphosphorylation. The DOR action is done by an inhibitory regulation of CDK5 and AMPK pathways. The attenuation of tau hyperphosphorylation is beneficial for the reduction of microtubule structure collapse and neuronal degeneration in AD conditions and helps to inhibit neuronal cell cycle reactivation and reduce neuronal apoptosis. Our novel findings suggest that DOR may be a new target for treating AD via the inhibition of tauopathies (Figure 6).

## Data Availability

The original contributions presented in this study are included in this article/supplementary material, further inquiries can be directed to the corresponding author.
